# The dynamics of stomatal closure of *Arabidopsis thaliana* determined by terahertz spectroscopy and a water transport model

**DOI:** 10.1038/s41598-025-20219-y

**Published:** 2025-09-23

**Authors:** Jochen Taiber, Jan Helminiak, Goretti G. Hernandez-Cardoso, Cornelius Mach, Alexander Jäckel, Oscar A. Naranjo-Montoya, Enrique Castro-Camus, Peter Ache, Rainer Hedrich, Martin Koch

**Affiliations:** 1 Faculty of Physics and Material Sciences Center, Philipps-Universität Marburg, Renthof 5, 35032 Marburg, Germany; 2 Julius-von-Sachs-Institut für Biowissenschaften, Universität Würzburg, 97082 Würzburg, Germany; 3 Centro de Investigaciones en Optica A.C., Loma del Bosque 115, Lomas del Campestre, 37150 Leon, Guanajuato Mexico

**Keywords:** Terahertz, Drought stress, *Arabidopsis thaliana*, Stomata, Plant sciences, Optics and photonics, Physics

## Abstract

Terahertz (THz) time-domain spectroscopy allows the detection of temporal changes of plant water content in vivo and non-destructively, for example over the course of the day or at the onset of drought stress. By studying a wildtype and a genetically modified variant of *Arabidopsis thaliana*, we observed significant differences in their dehydration dynamics. For a better understanding of the underlying processes, we modelled this behaviour with a simple rate equation model, compared the results with the experimental data and correlated our model with the biological regulatory mechanisms. In particular, under drought stress, we found an almost three times ($2.80\pm {}0.51$) higher maximal stomatal opening in the mutant than in the wildtype. Over the course of the day, the degree of stomatal opening shows an exponential decrease with a half-life $t_{1/2}$ of $12.3\pm {}$2.6 h in the wildtype and $3.4\pm {}$0.8 h in the mutant.

## Introduction

The development of stomata and stomatal control was a decisive step in the evolution of plants: Being able to dynamically adjust the opening state and thus the permeability of the epidermis makes it possible to adapt the water transport within the plant^1^. In this way, vascular plants were able to colonise a wide variety of habitats with the most diverse conditions^2^.

Under ideal irrigation conditions, the plant opens its stomata during daylight in order to be able to take up $\hbox {CO}_2$ for fixation via photosynthesis^3–5^. However, stomatal opening is associated with water loss due to the exchange of water vapour. Closing of stomata reduces leaf evaporation and enables survival under extended drought periods, although photosynthesis is limited under these conditions. The underlying regulatory process is based on a complex biochemical signalling chain.

In general, water transport in plants takes place unidirectionally from the soil to the roots and then to the leaves. Here, the water is released into the environment as water vapour via the stomata. About 97% of the water uptaken is lost through transpiration and not used for growth or biochemical processes^6^. The driving force for this movement is the water potential, which under normal conditions decreases constantly from the soil to the stomata and the surrounding atmosphere^6,7^. The water release is thus strictly controlled by the opening width of the stomatal pores.

Under drought conditions, the hormone abscisic acid (ABA) is synthesized, which initiates stomatal closure. In this process, ABA binds to a receptor that inactivates a PP2C-type phosphatase, which prevents the autophosphorylation of the protein kinase OST1 and other kinases. Phosphorylated OST1 can in turn phosphorylate the anion channel SLAC1 and thus activate it^8^. This is the initial step for an ion and water transport chain that leads to a volume decrease of the guard cells and finally results in stomatal closure^9^.

The opening state of the stomata in turn has a direct influence on the amount of water inside the plant leaf. Due to the fact that electromagnetic radiation in the THz range is strongly absorbed by water, THz spectroscopy is a very sensitive technique to measure changes in the water content of plants as shown in previous publications^10–16^. An advantage of THz spectroscopy over conventional methods, such as gravimetry^17,18^ or osmotic potential measurements^19,20^, is that measurements can be performed *in vivo*, non-destructively and in a completely contactless fashion such that the plant remains unperturbed during the entire measurement period. It is also a direct measurement method, as the measured signal depends on the number of water molecules in the beam path eliminating the need for debatable assumptions. This makes this technique particularly suitable for time-resolved studies of the regulatory behaviour of the water balance of plants^12^.

In our experiments, we compare the dehydration of wildtype *Arabidopsis thaliana* (Ler) and a mutant with defective protein kinase OST1, in other words, with defective stomatal response to drought stress (*ost1-2*,^21^). We present the first study including 20 plants in which the entire in-vivo water dynamics is monitored with minimal disturbance of the plants during the study. In addition, we model the drying process: First, we set up a water transport model based on biological process assumptions. We then select the relevant parameters including their time dependence describing the observed dynamics and vary them so that a good agreement between the model and each measurement is achieved. Finally, we interpret the obtained results in terms of their biological meaning.

## Results

### Comparison of wildtype and mutant drying behaviour using THz spectroscopy

**Fig. 1:**
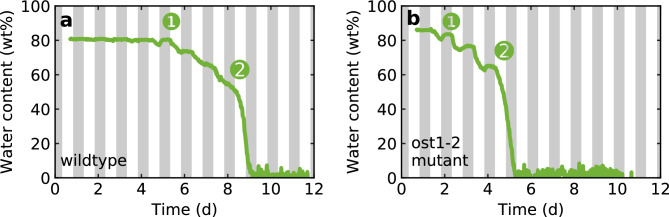
Drying process of (**a**) wildtype and (**b**) *ost1-2* mutant of *Arabidopsis thaliana*. We show the water content over a period of 11 days. The white background indicates the illuminated periods (12 h), the grey background the non-illuminated periods (12 h).

To motivate the following discussion, we present two exemplary measurement curves of the drying process of *Arabidopsis thaliana*, one for the wildtype (Fig. 1a) and one for the *ost1-2* mutant with a defective stomatal regulatory mechanism (Fig. 1b). Irrigation was stopped at time $t=-2.5\,\textrm{d}$. The ideal water status corresponds to the initial value of 80–85 wt%. The plots show that the wildtype can maintain this water level longer than the mutant. The time at which the water level is significantly reduced for the first time, starts three days earlier for the mutant. In the first night after this first water reduction, both wildtype and mutant can recover completely. After that, which is marked here with ❶, no complete recovery to the initial value can be observed for both plant types. Instead, wildtype and mutant lose water significantly thereafter, especially under illumination. After the water content has dropped below an average threshold of about 50 wt% (time ❷), no more water accumulation over the dark periods takes place. The leaf dries out completely within less than 24 hours. As a result of the earlier onset of desiccation in the mutant, the final state, i.e. complete drying of the measured plant leaf, also occurs earlier in the mutant than in the wildtype.

Particularly interesting, however, is the different behavior during the drying phase. For both plant types, the water loss is more severe in the illuminated periods than under non-illuminated conditions. In the mutant, however, this behavior is much more pronounced: Especially with illumination, the plant evaporates water to a large extent. These strong “day-night oscillations” were clearly visible in all *ost1-2* mutant plants measured which excludes random causes.

### Modelling the drying process

**Fig. 2:**
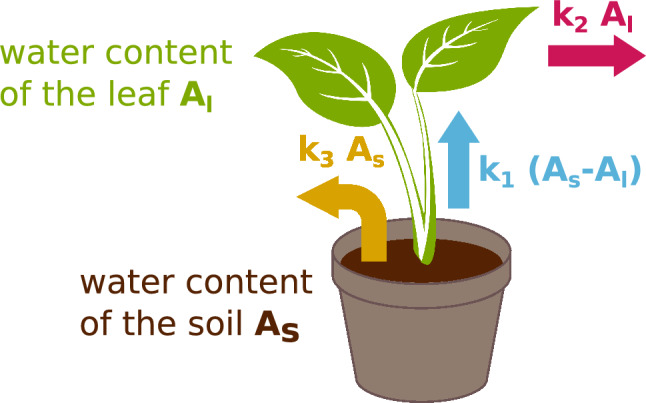
Modelling parameters: water contents $A_\textrm{l}$ and $A_\textrm{s}$ and water transfer rates with weighting factors $k_1$, $k_2$ and $k_3$.

For a better understanding of the underlying regulatory processes and to better quantify them, we present a compartmental model to describe the drying phase of a plant. First, we assume that the water in the sample system can be situated in two locations: in the soil and in the leafs. The water content $A_\textrm{s}$ of the soil in the pot and the water content $A_\textrm{l}$ of the leafs can be changed by two different processes each, which are characterized by water transfer rates (see Fig. 2).

The water transfer from the soil to the leaf is assumed to be proportional to the water content gradient, which is the difference of water content between the soil and the plant. The leaf gains water by drawing it from the soil at a rate of $k_1 \cdot (A_\textrm{s} - A_\textrm{l})$. On the other hand, it loses water through evaporation from the stomata which happens at a rate $k_2 \cdot A_\textrm{l}$. Likewise, the soil looses water by direct evaporation at a rate $-k_3 \cdot A_\textrm{s}$. By combining all these loss and gain rates, we can construct the following system of differential equations

**Fig. 3:**
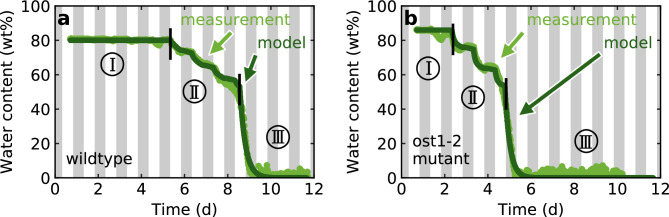
Modelling results (dark green) for the drying process of (**a**) wildtype and (**b**) *ost1-2* mutant of *Arabidopsis thaliana*. The measured values are shown in light green.

The time-dependent factors $k_i$ weight the corresponding rates: The factor $k_1$ modulates the water transfer from the soil to the plant leaf. The factor $k_2$ weights the water loss from the leaves, in other words, it represents the transconductance. Additionally, water evaporates from the soil for which the weighting factor $k_3$ is used. With this set of differential equations, the water dynamics of the plant-soil-system are modelled and the resulting solution is used to fit the experimental data. The results for exemplary drying phases of the wildtype and the *ost1-2* mutant of *Arabidopsis thaliana* are shown in Fig. 3. Here one can see that our model is able to describe the drying process qualitatively well in all temporal phases. Each fit parameter will be explained in the following paragraphs.

1 $$\begin{aligned} & \frac{\textrm{d}A_\textrm{l}}{\textrm{d}t} = k_1 \cdot (A_\textrm{s} - A_\textrm{l}) - k_2 \cdot A_\textrm{l}, \end{aligned}$$

2 $$\begin{aligned} & \frac{\textrm{d}A_\textrm{s}}{\textrm{d}t} = -k_1 \cdot (A_\textrm{s} - A_\textrm{l}) - k_3 \cdot A_\textrm{s}. \end{aligned}$$

**Fig. 4:**
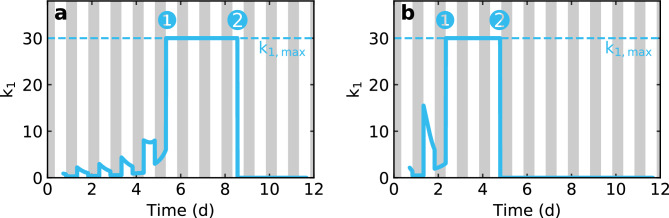
Modelling results for the weighting factor $k_1$ in the drying process of *Arabidopsis thaliana*. As long as the plant can maintain its optimum water content, the time course of $k_1$ is determined by the evaporation of the plant. However, if this is no longer the case due to the decreasing soil water content, $k_1$ reaches its maximum value $k_{1,{\textrm{max}}}$ (❶). This is identical for both the wildtype (a) and the mutant (b), which shows that the mutation does not cause any change in the processes that are decisive for the water transfer between roots and leaves. If the water potential of the soil falls below the critical threshold (permanent wilting point ❷), the plant can no longer take up water and $k_1$ drops to zero. In the wildtype, this happens at a later point in time than in the mutant, but at the identical water potential of the soil.

The factor $k_1$ is mainly dominated by a step-function-like water transfer at the onset of drought stress. The full time course is shown in Fig. 4. In detail, it can be explained as follows: First, the plant maintains its optimum water content despite water loss through evaporation by drawing water from the soil (until ❶) against a constant resistance. $k_1$ results from the differential equation 1 in the equilibrium state

3 $$\begin{aligned} k_1 = k_2 \cdot \frac{A_\textrm{l}}{A_\textrm{s} - A_\textrm{l}}. \end{aligned}$$

However, this resistance is decisive for the relevant phase II (see Fig. 3), because it determines the maximum possible water transfer, characterised by $k_{1,{\textrm{max}}}$. It is due to the retention of the soil and results physically from gravity, adhesion and cohesion. The equality of this value for both the wildtype and the mutant confirms that there is no difference in water transfer between roots and leaves.

If the permanent wilting point is reached (❷), which means that the water potential of the soil falls below a critical threshold, a regular water supply is no longer possible^22–25^. This interruption of the transpirational pull happens within a very short time period, which justifies the assumption of a step-like function of $k_1$. It is important here that the described threshold is identical for wildtype and mutant and occurs at the identical soil moisture content which is proved by gravimetric measurements.

For the factor $k_3$, which determines the evaporation rate from the soil into the air, we assume a constant value that is independent of the illumination, in order to reduce the number of fit parameters and to obtain a model that is as simple as possible. This assumption has also been experimentally verified by carrying out gravimetric comparative experiments (see Supplementary Fig. S5 online). In these experiments the evaporation from the soil without plants over several lighting cycles was measured. The very minor differences that occur can be justified by the very small soil surface of the pots that we used exposed to light and air.

**Fig. 5:**
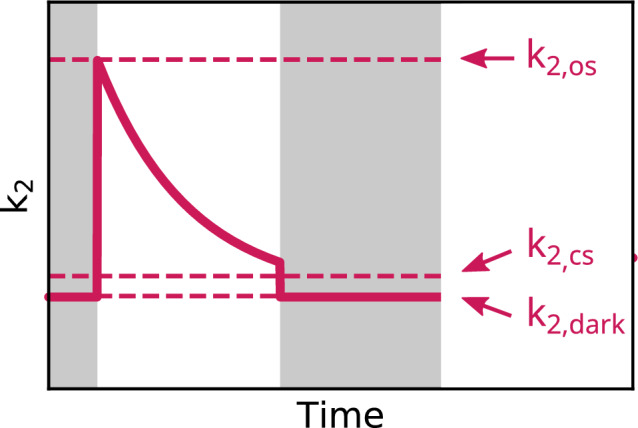
Definition of $k_2(t)$ by parameters.

The parameter $k_2$, which describes the transconductance, must show a clearly different dynamics for the wildtype and the mutant, revealing the defective regulatory pathway in the latter. Both plants open their stomata upon exposure to light (light-induced pathway)^21^, resulting in a large increase of leaf vapour exchange (see Fig. 5). Under drought stress, the plant now begins to close its stomata in order to reduce the water loss through evaporation.

Thus, we postulate that the parameter $k_2$ is described by an exponential decay during the bright periods

4 $$\begin{aligned} k_2(t) = (k_{2, \textrm{os}} - k_{2, \textrm{cs}}) ~ e^{-t/\tau {}} + k_{2, \textrm{cs}} \end{aligned}$$

and by a constant during the dark periods

5 $$\begin{aligned} k_2(t) = k_{2, \textrm{dark}}. \end{aligned}$$

**Fig. 6:**
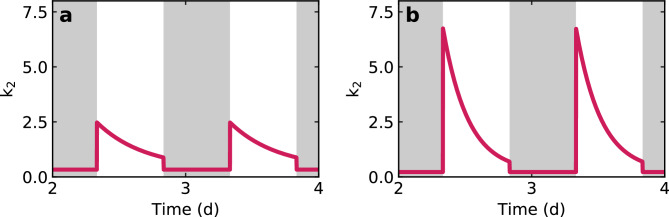
Modelling results for the weighting factor $k_2$, which describes the water loss of the leaf in the drying process of (**a**) wildtype and (**b**) *ost1-2* mutant of *Arabidopsis thaliana*. In the mutant, it reaches much higher values than in the wildtype.

This time dependency is determined by three fit parameters: The initial value $k_{2, \textrm{os}}$ with open stomata at the beginning of illumination, the value $k_{2, \textrm{dark}}$ without illumination and the time constant $\tau$ describing the exponential decay of the $k_2$ curve during the illumination phase. The final value $k_{2, \textrm{cs}}$ with closed stomata at the end of the illumination period is linked to $k_{2, \textrm{os}}$ via the constant factor $\Gamma _{\mathrm {bright-dark}}$ comparing the bright and dark conditions

6 $$\begin{aligned} k_{2, \textrm{dark}} = \Gamma _{\mathrm {bright-dark}} \cdot k_{2, \textrm{cs}} \end{aligned}$$

which is determined empirically. This takes into account that the water loss with closed stomata is still higher in illuminated conditions than in non-illuminated which will be discussed in more detail later. The entire $k_2$ dynamics for both wildtype and *ost1-2* mutant are shown in Fig. 6.

In order to provide a solid basis for our assumption, we have tested various alternative functions for the progression of $k_2$ over time (see Supplementary Fig. S2 and S3 online), corresponding to different physiological explanations. Due to these tests, we are able to show that the measurement results are only approximated sufficiently well with the assumption of an exponential decay which is typical in nature. Furthermore, it should be noted that the behaviour described here is only valid in the phase of existing drought stress, in other words when the water supply from the soil is insufficient. With optimal water supply, the plant tries to keep the stomata open for as long as possible.

**Fig. 7:**
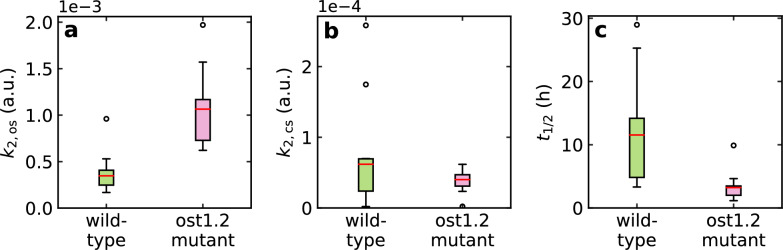
Obtained fit parameters for 20 individual plants. (**a**) The $k_{2, \textrm{os}}$ value for the mutant, which determines the maximum stomatal opening, is significantly higher than for the wildtype. By contrast, the minimum stomatal opening during illumination, indicated by $k_{2, \textrm{cs}}$, shows similar values for both wildtype and mutant (**b**). (**c**) The half-life $t_{1/2}$ for the exponential decrease in transpiration during the course of the day is higher for the wildtype than for the mutant (partly overlapping value ranges) which is also an effect of the rapid stomatal closure capability via the ABA signalling pathway.

Figure 7a shows that the water loss at the beginning of the illumination period is significantly higher in the mutant than in the wildtype. However, the value drops to the same final level over the course of the day (Fig. 7b). The half-life $t_{1/2}$ (Fig. 7c) again shows a significant difference between wildtype and mutant which is probably also due to the higher degree of opening at the start of illumination. The ratio between $k_{2, \textrm{os}}$ and $k_{2, \textrm{cs}}$ therefore serves as a characteristic parameter for the influence of the regulatory mechanisms on the water balance of the plant.

A look at the statistical significance: The parameter $k_{2, \textrm{os}}$, indicating the maximum stomatal opening, differs significantly at the 0.01 level between wildtype and mutant. The same applies for the half-life $t_{1/2}$ of the exponential closure of the stomata, but no significant difference can be found for $k_{2, \textrm{cs}}$, which indicates the minimum stomatal opening during illumination.

## Discussion

Our measurement technique provides a direct way to determine the hydration status of a plant over time without the need of fundamental assumptions, as is the case with indirect methods. Noteworthy is the detailed data set with 20 individual analyses. The behaviour described here can be seen in all measurement series carried out, which provides a solid basis for the conclusions drawn. By modelling the dehydration process, we improve the understanding of the plant mechanisms under drought stress. Our simple approach describes the experimental data very well, which indicates that transpiration is by far the dominant driving force for water uptake from the soil and transport. Further interactions between root/xylem and xylem/leaf are of course also of interest, but do not seem to play the major role in the net approach presented here. In detail: We can divide the drying process into three different phases:

- In the first phase (Fig. 3: phase I), the plant maintains its optimal water content in the leaves, although the water supply through the soil is already severely limited. This is only possible by active regulation of stomatal opening and thus the gas exchange. It can be seen by the fact that this phase lasts only about four days in the *ost1-2* mutant, whereas the water content remains at the optimal level for about seven days in the wildtype, calculated from the stop of irrigation.
- In the second phase (Fig. 3: phase II), the plant starts losing water significantly as the water supply from the soil is very low due to decreasing water potential. As a result of the light induced stomatal opening, the water loss is higher during illumination than in non-illuminated periods and is significantly higher in the *ost1-2* mutant than in the wildtype due to the flawed stomatal closure. At the beginning of this phase, the water content increases again under non-illuminated conditions as a consequence of the lower evaporation and the still present water supply from the soil. This nighttime moisture recovery appears more pronounced in the mutant due to the higher water loss under illumination. But towards the end of the phase, this is no longer the case and the plant also loses water under non-illuminated conditions. These day-night-oscillations are the characteristic feature of the plant’s dehydration behaviour.
- In the third phase (Fig. 3: phase III), the water transport to certain leaves is blocked. In addition to excessive soil water retention, further explanations include the occurrence of cavitation and embolisms^26–28^ after reaching the turgor loss point^29^. The water loss in this phase is even higher than with fully opened stomata, which indicates structural damage in the plant. Abscission or apoptosis processes to stop water loss through the leaves and to protect roots and stem may also play a role^6,30^.

Having a closer look at the modelling, the evolution of $k_1$ has to be mentioned. A good agreement with the results is obtained only by applying a discontinuous step (see Fig. 4a). It can not be obtained if the drop occurs softly over a longer period of time. As can be seen in Fig. 3, once the plant has fallen below a water content of about 50 wt%, the remaining water in the leaves rapidly decreases, much faster than could be explained by mere evaporation under unchanged conditions. This suggests that soil retention brings water uptake to an abrupt halt and supports the hypothesis of occuring embolisms or even apoptosis or abscission processes.

Regarding the stomatal behaviour: In the first two phases, there is a strong agreement between the model and the measured values if we assume an exponential decay for the water loss of the leaves with two different initial levels $k_{2, \textrm{os}}$, one for the wildtype and one for the mutant. The latter opens its stomata more strongly than the wildtype at the onset of illumination.

A key role is played by different rates of the processes that lead to stomatal closure. Interestingly, wildtype and mutant differ in the half-life $t_{1/2}$ of the exponential decrease in evaporation, which is 12.3$\pm {}$2.6 h in the wildtype and 3.4$\pm {}$0.8 h in the mutant. Both times are relatively long, which suggests that they do not describe a direct response to ABA production. The described ABA induced signaling via OST1 occurs on a very short time scale within several minutes and is not directly visible in the terahertz data as we measure the total water content in the leaf and not the inflow or outflow. Changes to these are accessible by turgor pressure measurements which the authors are showing in a different publication^9^. However, our focus in this study is on the change in water content in the leaf of the plant.

It is relevant to point out that in the *ost1-2* mutant, only the ABA induced signaling pathway via OST1 is disrupted. Stomatal regulation by light or CO$_2$, for example, is largely unrestricted^9,21^. In general: At the end of the non-illuminated period, the plant’s energy stores are relatively empty. The main focus is therefore on obtaining energy through photosynthesis. In order to absorb as much CO$_2$ as possible, both *Arabidopsis* variants open their stomata at the abrupt onset of light as wide as possible, which is accompanied by increased water loss. During the course of the day, the stores, e.g. in the form of starch, are refilled and reducing the water loss is prioritised more strongly again. While the wildtype reacts to water shortage as a criterion for regulating its water loss and prioritises this regulation, the mutant only follows pure energy demand management. This could explain the exponential course and the differences between wildtype und mutant. Dittrich et al.^31^ and Merilo et al.^32^ measured the gas exchange to show that deactivating the relevant ABA receptors has a significant influence on transpiration, but it is not exclusively dependent on these receptors and stomatal closure can still occur. A response to drought stress is, to a limited extent, therefore also possible via ABA independent pathways such as reduced hydraulic turgor pressure^2,33,34^ or low humidity^31,32^. The latter presumably also plays a role in this experiment due to the relatively low air humidity values.

Having a look at Fig. 7, which shows the values for the fit parameters of all evaluated plants: Comparing the values of the parameter $k_{2, \textrm{cs}}$ that describes the minimum water loss from the leaf under illumination, no significant difference can be found. This allows the conclusion that the mutation does not influence the minimum evaporation when the stomata are closed.

The influence of the mutation, however, is visible in the strongly differing maximum degree of stomatal opening and shows the intact or impaired regulation of the stomatal opening via the ABA pathway. It also seems likely that the wildtype does not fully open its stomata in acute drought stress which is another reason for the lower initial level of stomata opening at the beginning of the illumination. Our experiments even allow us to quantify the difference in the degree of opening: This is 2.80$\pm {}$0.51 times higher in the mutant than in the wildtype.

In order to achieve a good agreement between the model and the measured data, it is necessary that the water loss rate, determined by the parameter $k_2$, is lower during non-illuminated conditions compared to illuminated conditions and closed stomata. This can be caused by incomplete closure, for example through restricted control of stomata that are still under development, or by water loss through other paths, such as cuticular transpiration or via hydathodes. This hypothesis is supported by the fact that the stomatal conductance is not zero even with closed stomata as already shown^35–37^. In our study, we therefore introduced the factor $\Gamma _{\mathrm {bright-dark}}$ in Eq. (6) and determined it empirically by optimising the agreement of our model with the measurement data. This shows that by far the largest proportion of total water loss occurs through the open stomata, see also^38–40^. Lawson and Cominelli, for example, both gives a portion of 95%^36,41^ which is in adequate good agreement with our empirically determined value of $\Gamma _{\mathrm {bright-dark}}$ of 92%. Growing conditions also play a role: If these are optimal, i.e. if there is high humidity, insensitivity to ABA can occur resulting in reduced stomatal closure.

In addition to the fundamental findings on the drought stress response described above, we were able to reduce the different dynamics in the water balance between the wildtype and the *ost1-2* mutant to crucial parameters. A possible application for this could be in the field of phenotyping: Investigations using technologies in the terahertz range could provide an alternative method to thermal imaging in mutant screening, as proposed by Merlot et al.^42^. In this way, the influence of certain gene modifications on the water balance of the plant can be elucidated with high-precision measurements. Especially in view of longer periods of drought due to climate change, both a better understanding of the drought stress response and a tool that helps with developments in enhancing drought stress tolerance are eminently important.

## Methods

### Plant samples

Two variants of *Arabidopsis thaliana* were investigated: Landsberg erecta (Ler) as wildtype and *ost1-2* as mutant^21,42^ (photos are shown in Fig. S4 online). The plants were cultivated in the lab in tubes with 34,0 g soil each under fully controlled growing conditions: photoperiod of 12h with illumination by 85-95 µmol m$^{-2}$ s$^{-1}$ white light (400 W, Bio Green Sirius X400), air-conditioner regulated temperature of 18 $^\circ$, which rises to 20 $^\circ$ when illuminated, and relative humidity of 32–45 %. For the measurements, care was taken to select plants with the most uniform leaf growth possible to avoid differences in water loss due to different leaf surface areas. To improve the statistics, three series of 15 plants each were recorded. The automatic fit procedure was performed for each individual plant. 20 fit results (10x wildtype, 10x mutant) with an R$^2$ value $> 0.99$ were then selected for further evaluation, see results in Fig. 7. This selection of the measurement series according to the goodness of fit was carried out because the automated fit procedure was not possible for all plants. Some plants died prematurely, but not from water stress, and do not reach the characteristic phase II of the day-night oscillations, while others show measurement errors due to insufficient fixation of the leaf. Our fit selection criterion therefore appears appropriate, as all plants with a normal drought stress phase are taken into account.

### THz time-domain spectroscopy

**Fig. 8:**
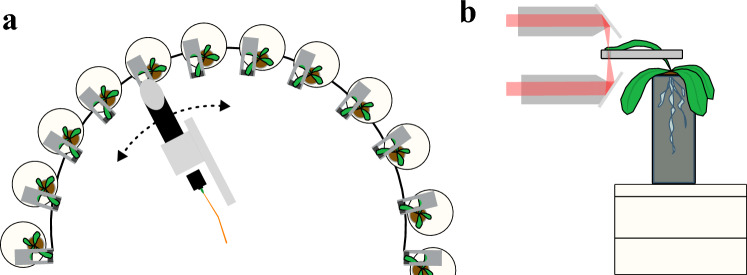
(**a**) Circularly moving measurement head for automated measurements of 15 plants. (**b**) Side view: the transmission beam path through a plant leaf is shown in red. The plant is positioned on a scale for additional gravimetric data.

All measurements were recorded using a THz time domain spectroscopy setup consisting of a 1550 nm fiber-coupled femtosecond laser (Menlo C-Fiber), a mechanical free space delay line and fibre-coupled photoconductive antennas (InGaAs/InAlAs multilayer structure). For long-term measurements of whole plant series, the system was fully automated, see Fig. 8: The antennas were mounted on a circularly moving arm of a goniometer, driven by a computer-controlled stepper motor to allow continuous measurements of 15 plants over a period of several weeks^11^.

### Measurements

All plants are arranged in the setup in such a way that one particular leaf is fixed by magnetic stripes and is thus available for transmission measurements. Over the entire duration of the experiment, each plant was always measured at the identical spot with a diameter of 3 mm at the mesophyll. Light conditions were 60–85 µmol m$^{-2}$ s$^{-1}$ white light (400 W, Bio Green Sirius X400) with a photoperiod of 12h. Care was taken to touch or shade the sheet only at the outermost edge to minimize the influence of the fixation. Before the start of a measurement series, irrigation of the plants was stopped to create drought stress. Each measurement series was then carried out for 8 to 14 days until each plant was completely dried out, with a measurement signal recorded every 25 min for each individual plant. Immediately before each measurement of a plant leaf, a reference measurement is taken to remove the influence of system fluctuations or ambient conditions.

### Signal processing (effective medium theory)

In each case, a complete THz time trace of 50 ps was recorded. By applying a Fourier transform, the signal is transferred into the frequency domain^43^. The experimental transfer function is calculated by dividing the measured transmission signal by the corresponding reference signal.

To extract the water content of the leaf from the measured THz signal, we apply an effective medium theory: Here, the plant leaf is considered as an “effective medium”, thus a composition of various substances. Instead of considering the complex structure, the leaf is described by the dielectric properties and the relative fractions of its individual components. In our case, we assume that the plant leaf consists of the three components air, water and dry material and then apply the Landau-Lifschitz-Looyenga model to calculate the resulting dielectric function of the leaf^13,44^

7 $$\begin{aligned} \root 3 \of {{\varepsilon }_{\textrm{res}}} = X_\textrm{water} \cdot \root 3 \of {{\varepsilon }_{\textrm{water}}} + X_\mathrm {dry~material} \cdot \root 3 \of {{\varepsilon }_{\mathrm {dry~material}}} + X_\textrm{air} \cdot \root 3 \of {{\varepsilon }_{\textrm{air}}}. \end{aligned}$$

**Fig. 9:**
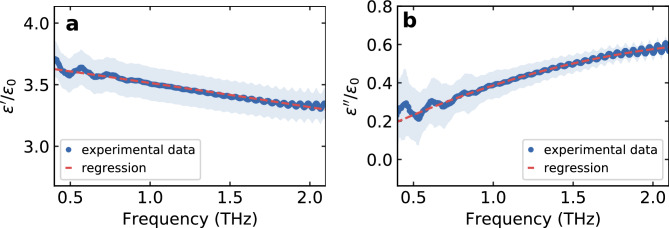
(**a**) Real and (**b**) imaginary part of the dielectric function of dry material of *Arabidopsis thaliana* in the terahertz band.

The dielectric function of water is approximated by the Double Debye model^45,46^, while that of the dry material is determined experimentally. To do this, the leaves of the measured *Arabidopsis thaliana* plants were removed and dried. They were then ground to a fine powder using a mortar and compressed into a tablet using a hydraulic press (100 µg material with an applied force of 30 kN over a period of 90 s). Subsequently, these tablets were processed using a THz transmission setup (HHI TeraWave^47^) under nitrogen atmosphere to avoid any influence of atmospherical water. The resulting dielectric function can be seen in Fig. 9 and can be fitted with *f* as the frequency in terahertz by

8 $$\begin{aligned} ~\varepsilon '=-0.01\,\textrm{THz}^{-2} \varepsilon _0 f^2-0.18\,\textrm{THz}^{-1} \varepsilon _0 f+3.7 \varepsilon _0~\textrm{and}~\varepsilon ''=-0.08\,\textrm{THz}^{-2} \varepsilon _0 f^2+0.43\,\textrm{THz}^{-1} \varepsilon _0 f+0.04 \varepsilon _0. \end{aligned}$$

Using Eq. 7, the theoretical transfer function can be calculated as explained in^13^. Then this is fitted to the experimentally obtained transfer function within the range from 0.1 to 0.3 THz, using the water content as the fit parameter $X_\textrm{water}$ and assuming a fixed ratio between $X_\mathrm {dry~material}$ and $X_\textrm{air}$ which was experimentally justified. It should be noted that the thickness of the plant leaf also plays a role: Since this changes during the drying process, a minor linear correction of the thickness based on experimental data was implemented in the evaluation program. This was designed by a combination of exemplary thickness determination from the terahertz signal itself using Fabry-Perot pulses^48,49^ and mechanical measurements using a micrometer screw, as an individual correction is too unstable due to the inhomogeneity and complexity of a plant leaf. In this way, the relative water content of the plant leaf can be obtained from the measured THz time domain signals. More detailed information about the application of the effective medium theory to biological samples can be found in^13,50–52^.

### Supportive measurements

In oder to quantify and to minimize the influence of the environment, the temperature, relative humidity, the amount of particulate matter and the CO$_{2}$ content of the atmosphere were permanently recorded. Individual scales for each plant, which are read out automatically, allow parallel recording of the weight changes and thus comparison with gravimetric data, which are in very good agreement with the THz data obtained.

## Supplementary Information

Supplementary Figures.

## Data Availability

The data that support the findings of this study are available from the corresponding authors upon reasonable request. Requests should be addressed to J.T.
