# Supplementary material for: The dynamics of stomatal closure of Arabidopsis thaliana determined by terahertz spectroscopy and a water transport model
Source: Sci Rep. 2025 Sep 23;15:32675. doi: 10.1038/s41598-025-20219-y (PMC12457618; doi:10.1038/s41598-025-20219-y)
Supplement: Supplementary file 1 — Supplementary Figures. [file 41598_2025_20219_MOESM1_ESM.pdf]

Supplementary Information: The dynamics of stomatal closure of *Arabidopsis thaliana* determined by terahertz spectroscopy and a water transport model

Jochen Taiber[1,*], Jan Helminiak[1], Goretti G. Hernandez-Cardoso[1], Cornelius Mach[1], Alexander Jäckel[1], Oscar A. Naranjo-Montoya[3], Enrique Castro-Camus[1,3], Peter Ache[2], Rainer Hedrich[2], and Martin Koch[1]

[1]Philipps-Universität Marburg, Faculty of Physics and Material Sciences Center, Renthof 5, 35032 Marburg, Germany
[2]Universität Würzburg, Julius-von-Sachs-Institut für Biowissenschaften, 97082 Würzburg, Germany
[3]Centro de Investigaciones en Optica A.C., Loma del Bosque 115, Lomas del Campestre, Leon, Guanajuato 37150, Mexico
[*]https://orcid.org/0000-0002-5884-1449

ABSTRACT

Terahertz (THz) time-domain spectroscopy allows the detection of temporal changes of plant water content *in vivo* and non-destructively, for example over the course of the day or at the onset of drought stress. By studying a wildtype and a genetically modified variant of *Arabidopsis thaliana*, we observed significant differences in their dehydration dynamics. For a better understanding of the underlying processes, we modelled this behaviour with a simple rate equation model, compared the results with the experimental data and correlated our model with the biological regulatory mechanisms. In particular, under drought stress, we found an almost three times (2.80±0.51) higher maximal stomatal opening in the mutant than in the wildtype. Over the course of the day, the degree of stomatal opening shows an exponential decrease with a half-life $t_{1/2}$ of 12.3±2.6 h in the wildtype and 3.4±0.8 h in the mutant.

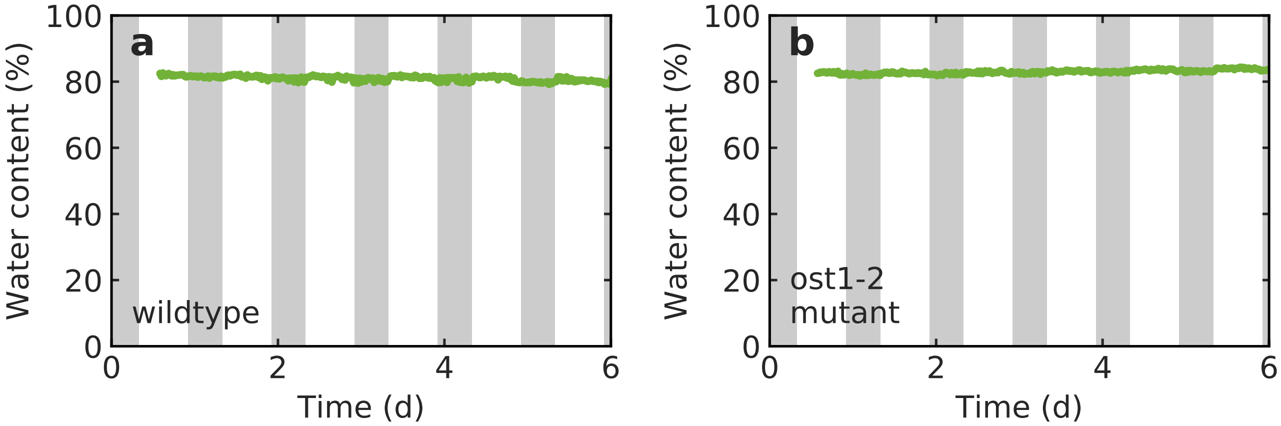

Figure S1. Exemplary measurement results of the water content of (a) wildtype and (b) *ost1-2* mutant of *Arabidopsis thaliana* before the onset of drought stress, i.e. with sufficient water supply from the soil. No relevant differences can be identified as drought stress-induced regulation of stomatal opening is not necessary here and instead the water uptake is not restricted. Significant differences between the two variants only become apparent under acute drought stress.

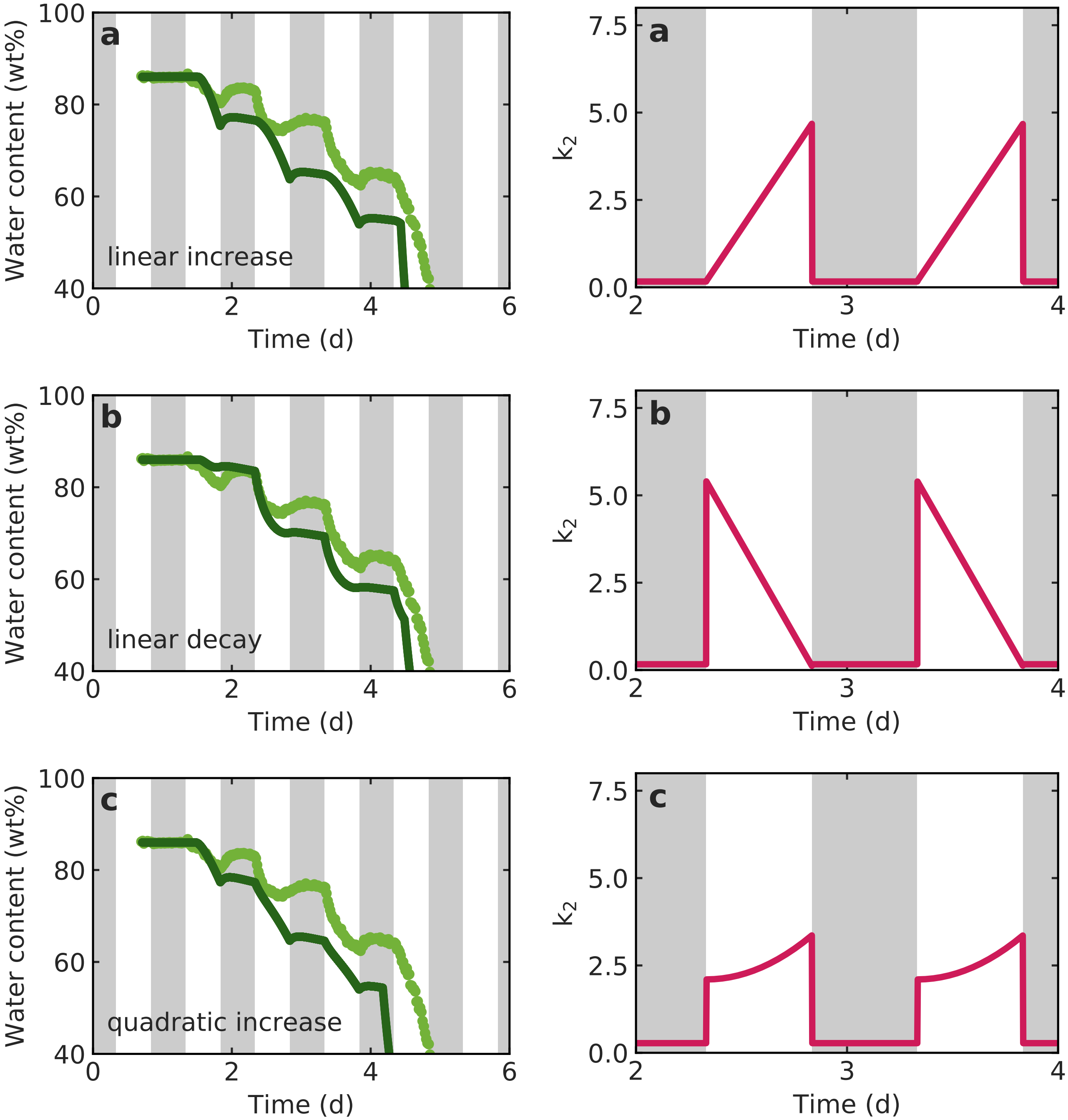

Figure S2. Various alternative functions to describe the time dependence of k_2: (a) linear increase, (b) linear decay, (c) quadratic increase. As an alternative to the assumed exponential behaviour of stomatal closing, we have tested other possible time dependencies which we have selected to describe various biological assumptions. However, the results show clearly that all alternative time dependencies lead to poorer fit results.

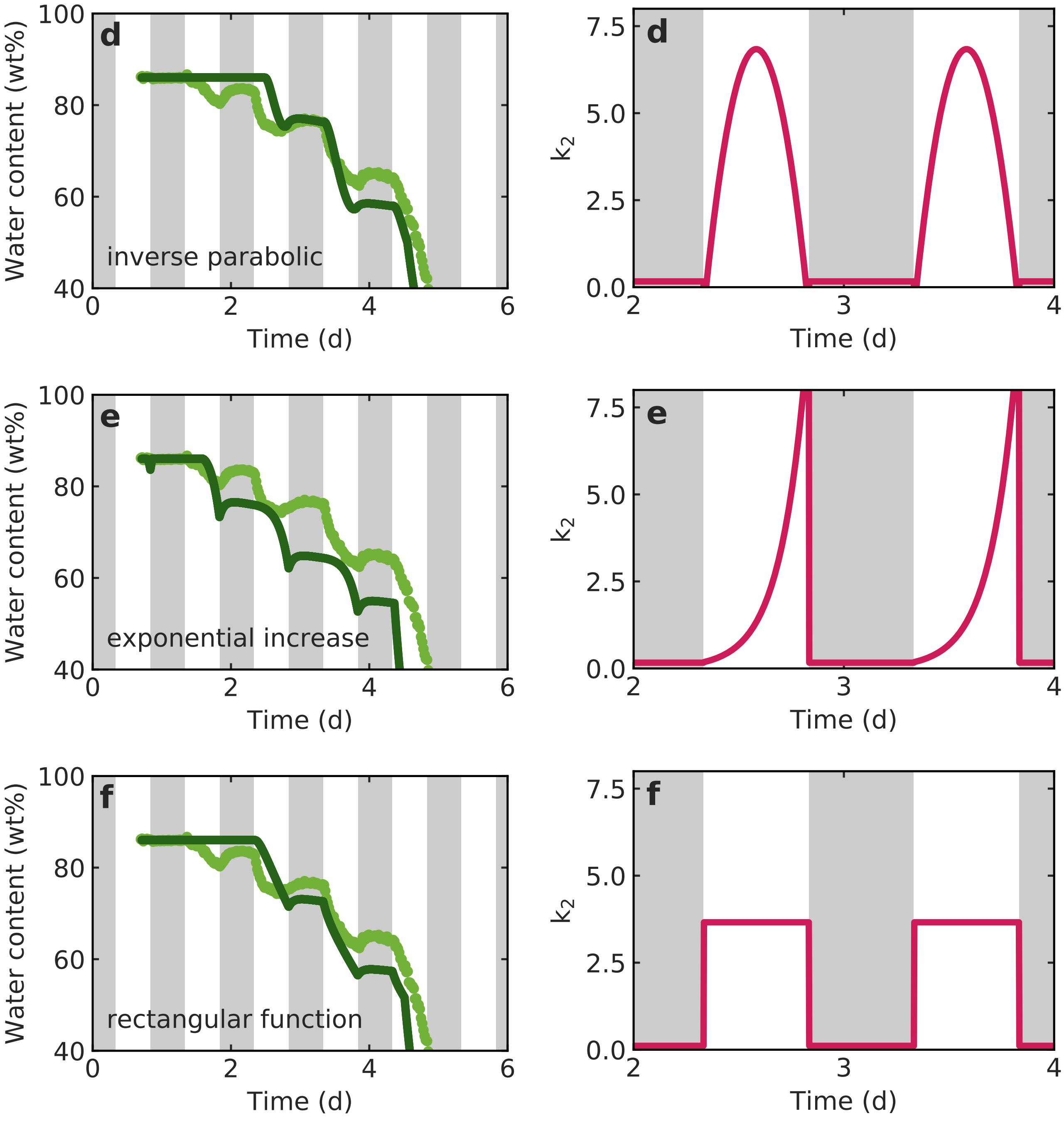

Figure S3. Various alternative functions to describe the time dependence of k_2: (d) inverse parabolic, (e) exponential increase, (f) rectangular function. As an alternative to the assumed exponential behaviour of stomatal closing, we have tested other possible time dependencies which we have selected to describe various biological assumptions. For example, an inverse parabolic course would describe a degree of stomatal opening increasing up to the 'middle of the day' with a maximum there. A curve corresponding to a rectangular function would also be possible, as this would follow the time dependency of the illumination. Even if a strong ABA-induced stomatal closure reaction in the wildtype is assumed, this would make sense, as this reaction takes place very quickly. However, the results show clearly that all alternative time dependencies lead to poorer fit results.

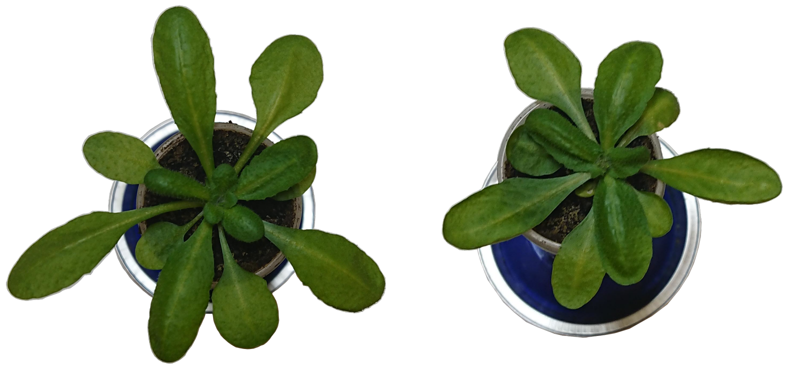

Figure S4. Wildtype (left) and *ost1-2* mutant (right) of *Arabidopsis thaliana*. The growth of both plant variants was very homogeneous, which makes differences in water loss due to different leaf surface areas negligible.

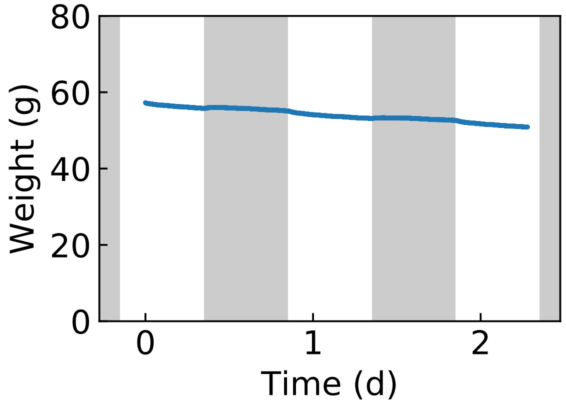

Figure S5. Weight data from a series of measurements in which the drying of soil was determined gravimetrically. We maintained the same conditions as in the plant experiments with regard to soil, pots, temperature, humidity and illumination. Differences in direct evaporation between illuminated (white) and non-illuminated (grey) periods can be neglected in our study. This is due to the fact that we used pots with a very small soil surface area exposed to light and air, as can be well seen in Fig. S4.

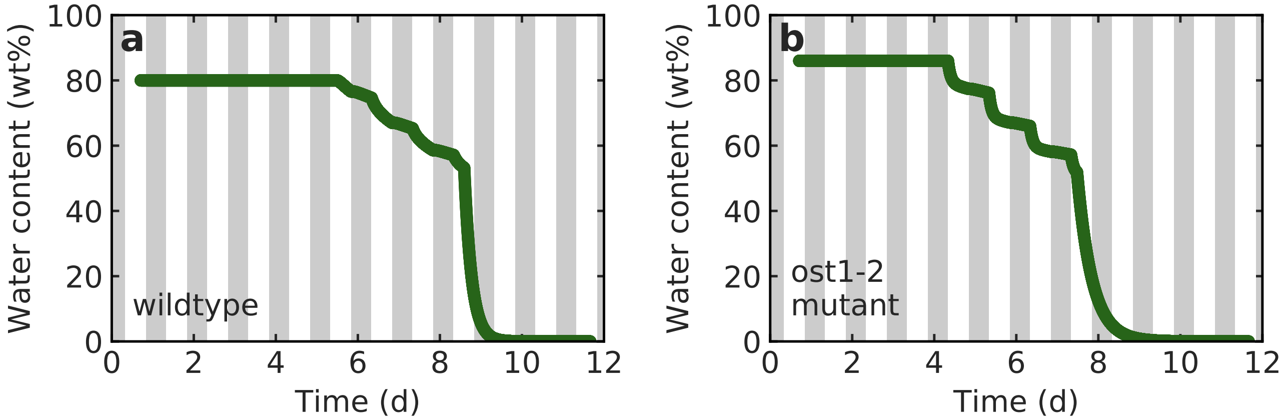

Figure S6. Averaged model of the drying behaviour of (a) wildtype and (b) *ost1-2* mutant of *Arabidopsis thaliana*. These curves result from the averaged fit parameters of the analysis of the 20 individual plants described in the main text. In the acute drought stress phase of the mutant, the pronounced day-night oscillations are clearly visible.
